# Quantification of Teicoplanin Using the HPLC-UV Method for Clinical Applications in Critically Ill Patients in Korea

**DOI:** 10.3390/pharmaceutics13040572

**Published:** 2021-04-17

**Authors:** Jaeok Lee, Eun-Kyoung Chung, Sung-Wook Kang, Hwa-Jeong Lee, Sandy-Jeong Rhie

**Affiliations:** 1 College of Pharmacy and Graduate School of Pharmaceutical Sciences, Ewha Womans University, Seoul 03760, Korea; leejo19@ewha.ac.kr; 2 Department of Pharmacy, College of Pharmacy, Kyung Hee University, Seoul 02453, Korea; cekchung@khu.ac.kr; 3 Department of Pharmacy, Kyung Hee University Hospital at Gangdong, Seoul 05278, Korea; 4 Department of Pulmonary, Allergy and Critical Care Medicine, Kyung Hee University Hospital at Gangdong, School of Medicine, Kyung Hee University, Seoul 05278, Korea; aikra@hanmail.net; 5 Department of Pharmacy, Ewha Womans University Mokdong Hospital, Seoul 07985, Korea

**Keywords:** teicoplanin, polymyxin B, HPLC-UV, internal standard, human plasma, clinical application

## Abstract

A high-performance liquid chromatography-ultraviolet detector (HPLC-UV) method has been used to quantify teicoplanin concentrations in human plasma. However, the limited analytical accuracy of previously bioanalytical methods for teicoplanin has given rise to uncertainty due to the use of an external standard. In this study, an internal standard (IS), polymyxin B, was applied to devise a precise, accurate, and feasible HPLC-UV method. The deproteinized plasma sample containing teicoplanin and an IS of acetonitrile was chromatographed on a C18 column with an acidic mobile phase consisting of NaH_2_PO_4_ buffer and acetonitrile (78:22, *v*/*v*) by isocratic elution and detection at 220 nm. The linearity was in the range 7.8–500 mg/L calculated by the ratio of the teicoplanin signal to the IS signal. This analytical method, validated by FDA guidelines with ICH Q2 (R1), was successfully applied to analyze the plasma samples of patients in the intensive care unit for treating serious resistant bacterial infectious diseases, such as those by methicillin-resistant *Staphylococcus aureus* and *Enterococcus faecalis*. The methods suggested the potential for use in routine clinical practice for therapeutic drug monitoring of teicoplanin, providing both improved accuracy and a wide range of linearity from lower than steady-state trough concentrations (10 mg/L) to much higher concentrations.

## 1. Introduction

Multidrug resistance is a growing threat to public health and is an emerging crisis in infection control and prevention [1]. Methicillin-resistant Gram-positive organisms have become a major cause of hospital- and community-acquired multidrug-resistant infections and are a constantly growing public health concern [2]. In the United States of America (USA), Europe, and some parts of Asia, the incidence of Methicillin-resistant Staphylococcus aureus (MRSA) has been monitored to establish a national database of the multidrug-resistant clones distributed worldwide [3]. In Korea, the MRSA rate was reported to be as high as 81% in tertiary hospitals and 40% in non-tertiary hospitals as early as 2009 [2].

Although vancomycin is widely used to treat MRSA and other methicillin-resistant Gram-positive organisms, increasing minimum inhibitory concentrations of vancomycin have been reported in Staphylococcus aureus, which are associated with vancomycin failure. To reduce the treatment failure of vancomycin, the doses of vancomycin have been increased but higher doses can result in increased nephrotoxicity [4]. Teicoplanin is one of the later introduced glycopeptide antibiotics with a comparable efficacy and a favorable safety profile in treating nosocomial infections by methicillin-resistant Gram-positive organisms in critically ill patients [5]. It further provides the susceptibility against vancomycin-resistant enterococci, except for VanB phenotype enterococci [6,7]. It has a longer half-life of approximately 100 vs. 10 h, which could be a substantial advantage compared to vancomycin [8,9]. Of note, regular monitoring of trough plasma levels of teicoplanin is not currently required in most clinical settings. However, as cases of teicoplanin treatment are for complicated infections, the continuation of a high-dose regimen with loading doses of 6 mg/kg twice daily for 48 h has been recommended to achieve target therapeutic concentrations of >10.0 mg/L by the second day of treatment [10]. Another study recommended the plasma trough concentration of >20.0 mg/L in the treatment of severe MRSA infections such as endocarditis with the first five loading doses of 10–12 mg/kg at 12-h intervals in patients with normal renal function [11]. Moreover, teicoplanin has recently reported the effectiveness against SARS-CoV-2 (COVID-19) in vitro by preventing viruses’ entrance into the cytoplasm with half-maximal inhibitory concentration (IC50) as 1.66 μM [12]. This indicates the need to monitor plasma concentrations, which requires an analytical method that is not only accurate and precise but also practical and clinically applicable. Currently, teicoplanin has been approved in Europe, Asia, and South America, but not yet in the USA [13].

As it has become important to maintain an optimal plasma concentration of teicoplanin, various analytical methods have been applied to determine the concentrations in the body fluids of patients, including fluorescence polarization immunoassay (FPIA), microbiology-based bioassays, and LC-tandem mass spectrometry (LC-MS/MS). However, the use is limited due to its high cost and lack of accessibility although some are highly sensitive and selective methods [14,15,16]. A high-performance liquid chromatography-ultraviolet detector (HPLC-UV) method is still preferable as a bioanalytical method in most clinical laboratories because of its analytical capability and universal accessibility. Most previously developed analytical methods have used teicoplanin as an external standard (ES) to quantify the antibiotic [17,18]. The use of an internal standard (IS) for quantitation of a target analyte by HPLC is pertinent to remove experimental errors associated with sample preparation procedures. The use of an internal standard when analyzing teicoplanin has been reported, including mephenesin [19], risticetin [16], polymyxin B [14,20], daptomycin [21,22], and piperacillin sodium [23]. Mephenesin has different chemical properties (hydrophobic) from teicoplanin, and risticetin, polymyxin B, and daptomycin were used for LC-MS/MS [14,16,20,21,22]. Recently, Wang et al. reported an HPLC-UV method using piperacillin as an IS in Chinese patients [23], but their method was not capable of monitoring over 100 mg/L of the antibiotic in patient plasma.

Therefore, we developed the accurate and precise analyzing method of teicoplanin to measure concentrations in human plasma using polymyxin B as an IS and successfully applied it to plasma samples of patients that were critically ill with suspected or confirmed infectious disease mostly caused by resistant pathogens.

## 2. Materials and Methods

### 2.1. Materials

The teicoplanin standard, polymyxin B sulfate standard, human plasma, and chloroform were purchased from Sigma-Aldrich (St. Louis, MO, USA). Tapocin^®^ injection was provided by HK innoN (Seoul, Korea). Polymyxin B was selected as the internal standard (IS) according to the previous study by Tsai [14] and it was found to have common structural features with teicoplanin. Acetonitrile (ACN) and sodium dihydrogen phosphate anhydrous (NaH_2_PO_4_) were obtained from Merck (Darmstadt, Germany). All reagents and solvents were commercially provided HPLC analysis grade.

### 2.2. Apparatus

The HPLC analyses were performed on an Agilent Technology 1260 infinity system (Agilent Technology Inc., Waldbronn, Germany) equipped with an Agilent ChemStation software Rev. B.04.03-SP2 (105), a binary pump, a degasser, an autosampler, a thermostatted sample reservoir, a thermostatted column compartment, and a UV-detector. A Capcell-pak C18 UG120 column (4.6 mm × 250 mm, 5 μm, Osaka Soda Co. Ltd., Osaka, Japan) was used as an analytical column. The mobile phase was composed of 10 mM NaH_2_PO_4_ (pH 1.61–1.63) and 100% ACN (78:22, *v*/*v*) with isocratic elution at pH of 2.0–2.1 using a flow rate of 1.0 mL/min. The sample reservoir and column oven were maintained at 4 °C and 25 °C, respectively. The analytes were detected using a UV detector with the wavelength set at 220 nm, and the injection volume was 25 μL.

### 2.3. Sample Preparation

Sample preparations to determine total teicoplanin concentrations were performed according to the previously developed method by Roberts et al. [18]. Teicoplanin and IS solutions were prepared with fresh ultrapure water immediately prior to use. A total of 200 μL of plasma containing teicoplanin and the IS (100 ng/L) was deproteinized with 400 μL of ACN. The aqueous supernatant (approximately 100 μL) was prepared as the final sample extract after rinsing with 600 μL of chloroform.

### 2.4. Validation

#### 2.4.1. Linearity; Lower limit of quantification (LLOQ)

The calibration curve with total teicoplanin concentrations in plasma (x) vs. the ratio of the analyte peak area (A2-2) to the IS peak area (B2) (y) was constructed for the patient samples using seven concentrations in the range of 7.81–500.0 mg/L. The calibration curve was validated over three different occasions. The LLOQ was defined as the lowest concentration on the calibration curve with a precision of ≤20% coefficients of variation (CV) and an accuracy of 80–120%. The signal to noise ratio at the LLOQ was no less than 5.

#### 2.4.2. Precision and Accuracy

The precision of the peak area ratios and the accuracy were determined at low (7.81 mg/L), middle (62.5 mg/L), and high (500.0 mg/L) concentrations. The intra-day (three different experiments) and inter-day (three different occasions) precision of both the retention times and the peak area ratios are presented as CVs (acceptable if ≤5%). The accuracy is presented as the percent recovery.

#### 2.4.3. Recovery and Stability

The recovery of the sample preparation method was estimated by dividing the peak area for the pre-spiked sample by the peak area for the post-spiked sample, multiplied by 100. The stability of teicoplanin and IS in human plasma was investigated at low and high concentrations in three different experiments. Short- and long-term stability was evaluated after storage at 4 °C for 36 h and −70 °C for 24 days. Freeze-thaw stability was assessed with three freeze-thaw cycles at −70 °C. The post-preparation stability was also examined at 4 °C for 24 h. Teicoplanin stability was expressed as the relative recovery (%) of the freshly prepared sample.

### 2.5. Clinical Application

The newly developed bioanalytical method for teicoplanin with IS was utilized to measure teicoplanin concentrations in the plasma of patients. Patients received teicoplanin (Tapocin^®^ injection) loading doses of 6 mg/kg intravenously every 12 h for 3 doses and a maintenance dose of 6 mg/kg intravenously every 24 h administered over 20–30 min. Five blood samples from each patient were obtained through a central line at a randomly assigned time schedule from 0.5 h prior to infusion, 0, 0.5, 1, 2, 4, 6, 12, and 24 h after the end of the infusion. At each time point, 8 mL blood was collected in heparinized tubes and centrifuged at 3000–4000 rpm for 10 min at 4 °C to obtain plasma aliquots. They were transferred into Eppendorf tubes and stored at −70 °C until analysis. This study protocol was approved by the ethics committee of Kyung Hee University Hospital at Gangdong (Goyang, South Korea) (IRB No. 2019-04-002, April, 2019).

## 3. Results and Discussion

### 3.1. Optimization of the HPLC-UV Method and IS

**Figure 1:**
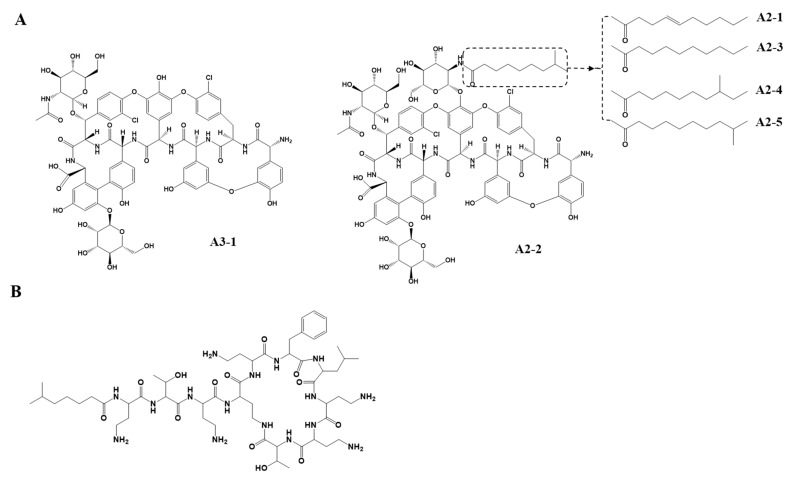
The structures of teicoplanin (**A**) and polymyxin B2 (**B**).

To develop a precise and accurate HPLC-UV method for the quantification of teicoplanin in human plasma, the parameters of the bioanalytical assay with IS were optimized. Initially, a simple LC method (10 mM NaH_2_PO_4_:ACN:methanol = 70:25:5 (*v*/*v*/*v*) (pH 2.1) as a mobile phase and UV detection with the wavelength set at 240 nm) without IS, as reported for a population pharmacokinetic study of teicoplanin [5], was utilized as a backbone to devise our novel analytical method with IS. This previously developed analytical method was modified to maximize the accuracy of quantitating teicoplanin in human plasma samples. Teicoplanin has six components of A3-1, A2-1, A2-2, A2-3, A2-4, and A2-5 (approximately 5.4, 2.8, 58.4, 5.4, 18.2, and 9.8%, respectively). In our experiments, we measured teicoplanin A2-2, which is the major isoform that makes up approximately 60% of the components. Its analysis is necessary for evaluating the concentration of teicoplanin in human plasma [24] (Figure 1A), because it shows stable reliability and validity during assays without interference while maintaining a simple sample preparation procedure.

First, the UV detector wavelength was set at 220 nm to optimize the sensitivity for detection of the major peaks of teicoplanin and polymyxin B (B1 and B2), which is consistent with the previously used UV wavelengths between 210 nm and 240 nm for teicoplanin [5,14,17,18] (Supplementary Figure S1A,B). Next, various conditions of the mobile phase, specifically pH and hydrophilicity, were investigated to eliminate any interference between the A2-2 teicoplanin (the major isoform) peak and the endogenous peaks of human plasma for optimal separation of each peak (Supplementary Figure S1C). Endogenous interferences were substantially attenuated by adjusting the mobile phase pH to an acidic condition, close to pH 2.0. Even with a change in the mobile phase pH from 2.1 to 5.2, no significant alteration in the retention times (RT) of teicoplanin or the IS peaks were observed; however, the IS peaks were substantially overlapped with the endogenous plasma peaks with an increase in the mobile phase pH (Supplementary Figure S2). Increased hydrophobicity of the mobile phase with a higher percentage of ACN (over 40%) produced overlapping peaks between IS and the endogenous substances of human plasma earlier than 3.5 min of RT in our pilot study; moreover, the major peaks of teicoplanin were not observed within 3 min. After evaluating a series of various mobile phase compositions, the final mobile phase for optimal chromatographic separation was determined to consist of 10 mM NaH_2_PO_4_ buffer and ACN (78:22, *v*/*v*) (Figure 1B). In addition, a possible effect of acetonitrile on teicoplanin and IS (sample preparation) was not considered in the present study, because the recovery rate of teicoplanin (A2-2) and IS (B2) were 104.7 and 98.5%, respectively (Supplementary Table S1). However, the effect of solvent on analyte recovery should be assessed in further studies.

**Figure 2:**
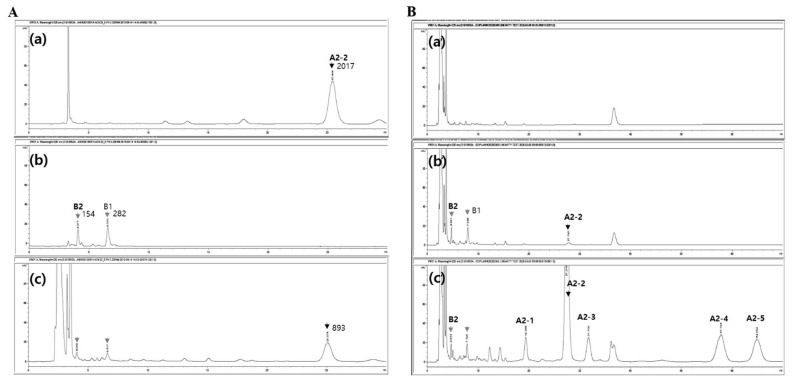
The elution of teicoplanin (A2-2) and IS (B2) with the optimized HPLC-UV method. (**A**) The chromatograms of (**a**) 100 mg/L teicoplanin solution without plasma, (**b**) 100 mg/L IS solution without plasma, and (**c**) teicoplanin and IS (each 50 mg/L) in human plasma. Numbers represented the indicated peak area. (**B**) The chromatograms of (**a’**) human plasma only (blank), (**b’**) teicoplanin (7.81 mg/L) and IS, and (**c’**) teicoplanin (500.0 mg/L) and IS in the plasma. Black and grey arrows indicate A2-2 and the major forms (B2 and B1) of IS, respectively.

The aforementioned optimized analytical method adequately detected the major peaks of teicoplanin and IS (Figure 2). The RTs of the major peaks of both were shifted and avoided the interferences; A2-2 at RT 10 min moved to RT 28 min, and the two peaks of IS at RT 3.5 min were separated at different RTs of 4 min and 7 min (Supplementary Figures S1 and S2). The two major peaks of IS eluted at RT 4 min and RT 7 min represented polymyxin B2 and B1, respectively [25,26]. In the repeated experiments, the peak of B2 showed more stable elution from the peak interference than that of B1. Therefore, the B2 peak was used for the quantification of teicoplanin A2-2. All the peaks were eluted by RT 60 min (Supplementary Figure S3). Teicoplanin and polymyxin B consist of multiple components, making the development of an appropriate quantitative analysis method challenging. In the present study, we detected all isoforms (A3-1:A2-1:A2-2:A2-3:A2-4:A2-5 = 5.4:2.8:58.4:5.4:18.2:9.8) for a possible TDM of all isoforms further after the peak of the major isoform of teicoplanin (A2-2). Our final optimized LC method adequately separated the major peaks representing each component of teicoplanin (six major peaks [24]) and polymyxin B (two major peaks [25]) in human plasma samples.

### 3.2. Validation of the Optimized Method: Specificity, Linearity, Sensitivity, Accuracy, Precision, Recovery, and Stability

Our newly developed, optimized HPLC-UV method was validated according to the US-FDA guidance with ICH Q2 (R1) [27]. The method validation was performed for specificity, linearity, sensitivity, accuracy, precision, and recovery. Teicoplanin A2-2 and polymyxin B2 were eluted at 27.5 min and 4.6 min, respectively, with a total run time of 70 min (Figure 2). The peaks for teicoplanin A2-2 and polymyxin B2 were free of interfering peaks. The LLOQ was 7.81 mg/L and the calibration curve showed linearity over the range of clinically relevant teicoplanin concentrations [28] (y = 0.1380x − 0.0888, coefficient of determination (r^2^) = 1.0000). Meanwhile, in the case of without IS, the linear regression differed from that with IS (y = 17.183x + 526.52, [r^2^] = 0.9949) and the lowest concentration point for the valid linearity was 125.0 mg/L, which supported the rationality of our method having IS in teicoplanin analysis. The HPLC-UV method using IS for teicoplanin analysis is seldom. One publication reported a practically available HPLC-UV method using piperacillin as the IS [23].

The intra- and inter-day accuracy and precision were assessed for the HPLC-UV method with IS. For the analytical method with IS, the within- and between-run accuracy and precision were in the range of 93.92–110.7% (accuracy) and 3.69–13.8% (precision), respectively (Table 1). These results are acceptable according to the regulatory guidance published by the FDA with ICH Q2 (R1). In terms of recovery, the mean ± standard deviation recovery of teicoplanin (A2-2) and IS (polymyxin B2) were 104.7 ± 14.8% and 98.47 ± 6.09%, respectively (Supplementary Table S1).

Compared to the previously developed and validated LC-MS/MS method, the LLOQ of our newly devised analytical method was high (0.32 mg/L vs. 7.81 mg/L) [14]. However, considering the steady-state trough concentrations (Css, min) of teicoplanin have been typically reported to be ≥10.0 mg/L in human plasma, the HPLC-UV method developed and validated in this study was adequate for the quantitative analysis of teicoplanin for our ongoing population pharmacokinetic study of teicoplanin [29]. While the LC-MS/MS technique provides many advantages such as higher specificity and sensitivity compared to the HPLC-UV method, its use in clinical practice is limited because it requires expensive instrumentation and highly skilled, trained personnel [28,30,31]. Moreover, the quantification range was wide with a concentration of up to 500 mg/L. This is beneficial as our clinical target is critically ill patients that have complex clinical characteristics including the prolonged high-dose administration of teicoplanin that depends on perfusion status, infection site, and multidrug-resistance, while most methods using LC-MS/MS or HPLC-UV are available to quantify up to 100 mg/L [14,20,22,23] or 200 mg/L [16].

In addition, ion suppression due to matrix effects of improperly prepared samples may exaggerate the errors in quantifying the analyte by LC-MS/MS [32]. Therefore, the development of a validated HPLC-UV method with adequate accuracy, precision, sensitivity, and selectivity is important for quantitative analysis, particularly regarding drugs in clinical practice, considering its ease of use and availability in most analytical laboratories.

The short-term, long-term, freeze-thaw, and post-preparation stability of the analytes (teicoplanin and IS) were investigated based on the peak areas of A2-2 and polymyxin B2, respectively (Table 2 and Supplementary Table S1). Overall, the stability (mean ± standard deviation) of teicoplanin and IS ranged from 96.19 ± 2.79% to 112.4 ± 5.12%, suggesting adequate stability of teicoplanin and IS in human plasma under the tested conditions of the analytical experiments.

### 3.3. Application to Patient Samples from the Ongoing Clinical Study

**Figure 3:**
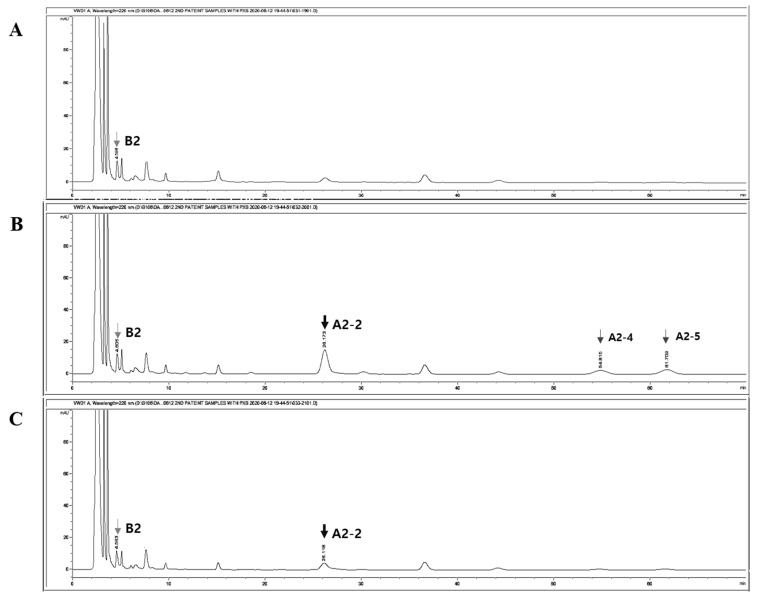
The chromatogram of teicoplanin (Tapocin^®^ injection) (A2-2) with IS (B2) in a patient’s 3 plasma samples. (**A**) Before teicoplanin administration. (**B**) At 1 h after the administration. (**C**) At 12 h after the administration. Light grey arrow indicated IS. Black bold arrow and dark grey arrows indicate A2-2, A2-4, and A2-5, respectively.

**Figure 4:**
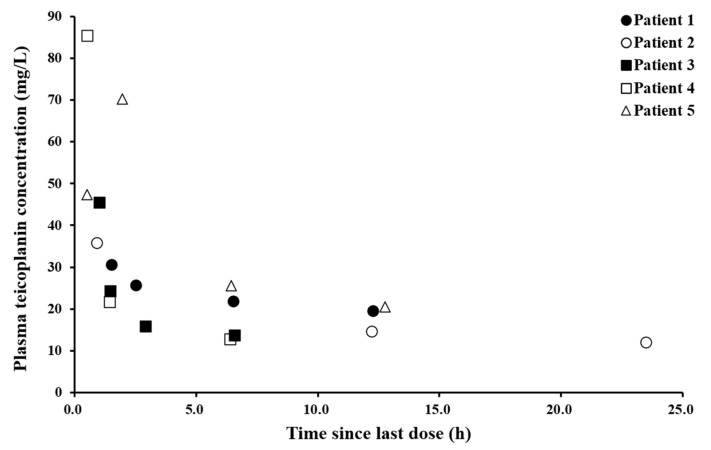
The plasma teicoplanin concentration–time since last dose curve from patients who were treated for methicillin-resistant Gram-positive organisms.

Our novel, optimized, and validated analytical method was successfully applied to 18 plasma samples from five patients for the determination of plasma teicoplanin concentrations (Figure 3 and Figure 4). The confirmation of the identity of teicoplanin in the sample is important. It can be performed by comparing spectra of the analyte using various applications, such as an HPLC-diode array detector (DAD) or LC-MS/MS. In the study, we monitored the peaks that appeared at retention time according to sampling time points after teicoplanin administration by UV-visible spectrometer instead (Figure 3). The presence of teicoplanin in patients’ plasma was confirmed by comparing the peaks of teicoplanin isoforms, A2-2, A2-4, and A2-5 (each area ratio = 1:0.32:0.39) appeared at 30 min (Figure 3B) after teicoplanin administration to those before the administration (Figure 3A). The peaks of A2-4 and A2-5 disappeared at 12 h (Figure 3C).

The teicoplanin (Tapocin^®^ injection) injected into patients was analytically identified as the same components with teicoplanin standard (Sigma) (Supplementary Figure S4). The plasma concentration of teicoplanin (Tapocin^®^ injection) ranged between 12.0 and 85.4 mg/L in our patient population, which indicates comparable limits of quantification. The concentration after the loading dose (at 24 h) reached approximately 12.0 mg/L, while the highest concentration was found at 0.5 h in the range of 47.3–85.4 mg/L. The variability among the subjects can probably be explained by the multiple factors that affect drug metabolism, such as clinical condition, renal functionality, administration of other medications, etc. Figure 4 shows the observed teicoplanin plasma concentration vs. time curve.

So far therapeutic monitoring of teicoplanin in a clinical setting is not routinely conducted due to its wide therapeutic range and positive safety profile. However, when it comes to the patients who required teicoplanin administration, most of them are critically ill and have complex clinical characteristics. They may require the prolonged or high-dose administration of teicoplanin depending on perfusion status, infection site, and multidrug-resistant organisms. Moreover, they may be exposed to unexpected or toxic ranges of plasma concentrations due to acute organ function changes, interaction with other concomitant multidrugs, or malnutrition status. There has been an emerging need for TDM in Korean patients, especially critically ill patients. In the present study, the analysis time for all isoforms eluted was long. Thus, it needs to consider applying gradient conditions which can provide a shorter elution time than the method in the text. Our efforts to quantitatively analyze teicoplanin using HPLC-UV can provide benefits in accessibility and affordability, and further in applicability allowing for therapeutic monitoring in the range of therapeutic concentrations.

## 4. Conclusions

We developed and validated a novel HPLC-UV method using IS (polymyxin B2) with high precision and accuracy for the quantitative analysis of teicoplanin in patient plasma. Using the newly developed HPLC-UV analysis, it would potentially allow for therapeutic concentration monitoring in plasma raging from lower than Css, min (10 mg/L) up to high concentrations (~500 mg/L). The quantitative analytical method newly devised in our current study based on an HPLC-UV technique may be useful for analytical and clinical purposes to determine teicoplanin concentrations in plasma from the receiving patients.

## Figures and Tables

**Table 1:** Intra- and inter-day accuracy and precision of teicoplanin (A2-2) with IS ^a^.

Concentration (mg/L) ^b^	Intra-Day (*n* = 9)		Inter-Day (*n* = 3)	
	Accuracy (%)	Precision (%, CV)	Accuracy (%)	Precision (%, CV)
7.81 (Low)	110.7 ± 15.3	13.8	93.92 ± 12.2	13.0
62.5 (Middle)	108.5 ± 9.44	8.70	106.2 ± 4.40	4.14
500.0 (High)	108.8 ± 10.4	9.58	103.5 ± 3.82	3.69

The values of accuracy, mean ± standard deviation (SD) CV; coefficients of variation. ^a^, Based on the ratio of teicoplanin A2-2 to polymyxin B2 signals. ^b^, Represents the concentration of the teicoplanin mixture.

**Table 2:** Stability of teicoplanin and IS (*n* = 3) ^a^.

Compound (Conc.) ^c^	4 °C, 36 h (%)	−70 °C, 24-d (%)	Freeze-Thaw (%)	Post-Prep. (%) ^b^
Teicoplanin (Low)	106.1 ± 13.1	110.0 ± 10.2	111.4 ± 6.26	103.5 ± 16.1
Teicoplanin (High)	103.3 ± 2.29	103.2 ± 0.62	112.4 ± 5.12	104.2 ± 11.9
IS (100 mg/L)	96.19 ± 2.79	112.2 ± 8.74	111.5 ± 7.36	

Conc., concentration; h, hour; d, day; IS, internal standard. Low conc., 7.81 mg/L; high conc., 500.0 mg/L; the values of accuracy, mean ± S.D. ^a^ Stability of teicoplanin and IS based on the peak area of A2-2 and polymyxin B2, respectively. ^b^ Post-preparation of teicoplanin and IS mixture, 4 °C, 24 h. ^c^ Concentration of teicoplanin or polymyxin B mixture.

## Data Availability

Data is contained within the article or Supplementary Material.

## Supplementary Materials

The following are available online at https://www.mdpi.com/article/10.3390/pharmaceutics13040572/s1, Figures S1–S4: Chromatograms of teicoplanin elution; Table S1: Stability of A2-2 and IS.
