# Supplementary material for: Quantification of Teicoplanin Using the HPLC-UV Method for Clinical Applications in Critically Ill Patients in Korea
Source: Pharmaceutics. 2021 Apr 17;13(4):572. doi: 10.3390/pharmaceutics13040572 (PMC8072975; doi:10.3390/pharmaceutics13040572)
Supplement: Supplementary file 1 [file pharmaceutics-13-00572-s001.zip › pharmaceutics-1154551-supplementary.pdf]

pharmaceutics

MDPI

Supplementary Materials: Quantification of Teicoplanin Using the HPLC-UV Method for Clinical Applications in Critically Ill Patients in Korea

Jaeok Lee , Eun-Kyoung Chung [2,3], Sung-Wook Kang, Hwa-Jeong Lee and Sandy-Jeong Rhie

A

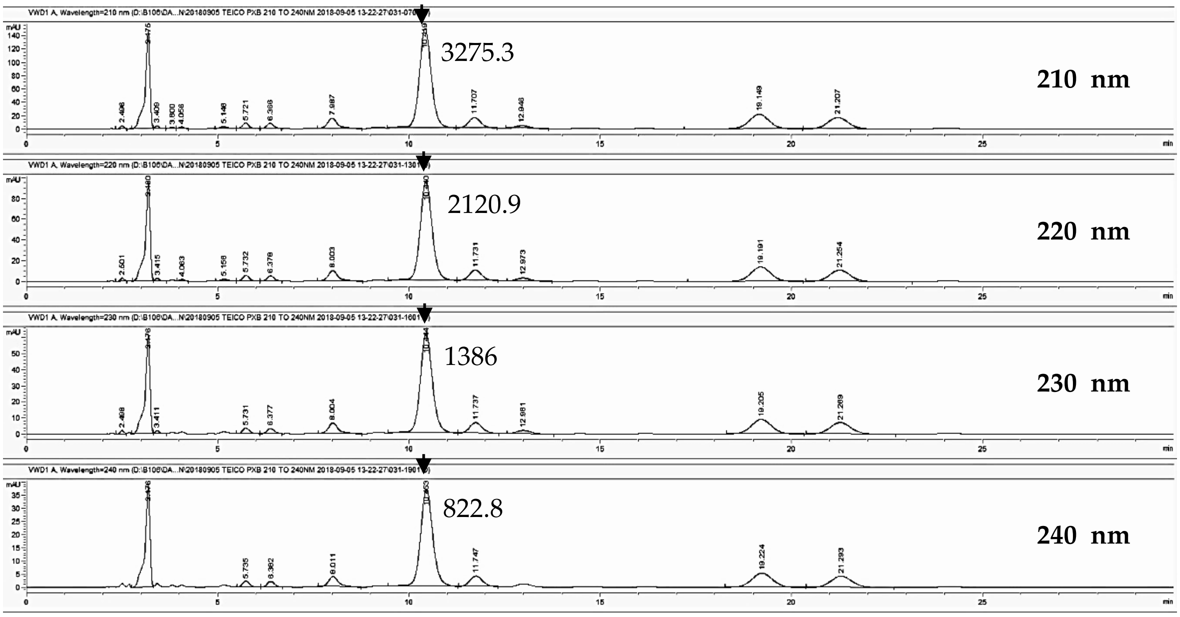

B

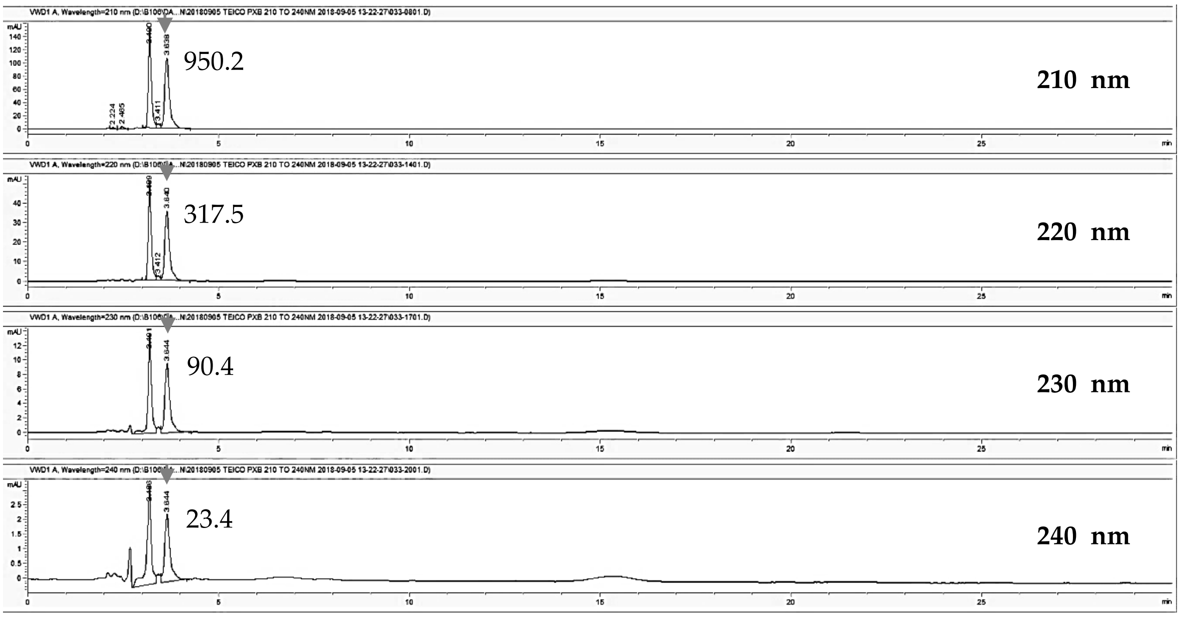

C

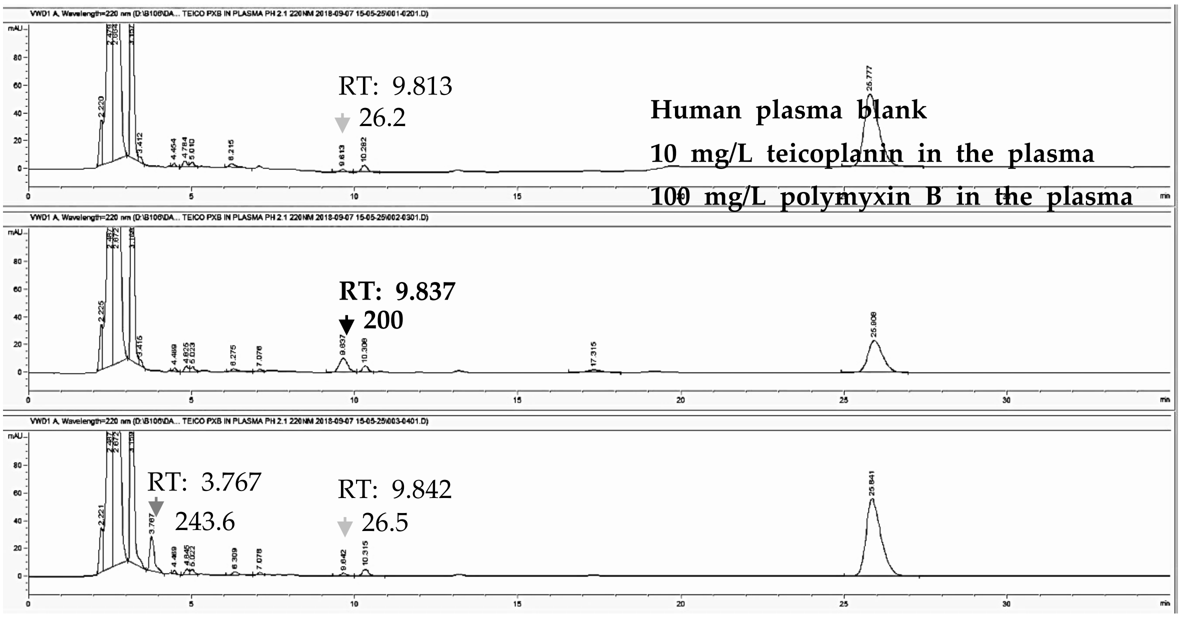

Figure S1. The sensitivity of the analyte detection by different UV wave lengths. (A) Teicoplanin (100 mg/L) peaks. Black arrows indicated A2-2, the major form of the antibiotic. (B) polymyxin B (100 mg/L) peaks. Grey arrows indicated B1, the major form. (C) The peaks of teicoplanin and polymyxin B1 in human plasma, at 220 nm detection. Light grey arrows indicated the endogenous peak. Mobile phase was 10 mM NaH_2PO_4:ACN:MeOH=70:25:5 (*v*/*v*/*v*), pH 2.0 for all three chromatograms. Numerical values represented the indicated peak area.

A

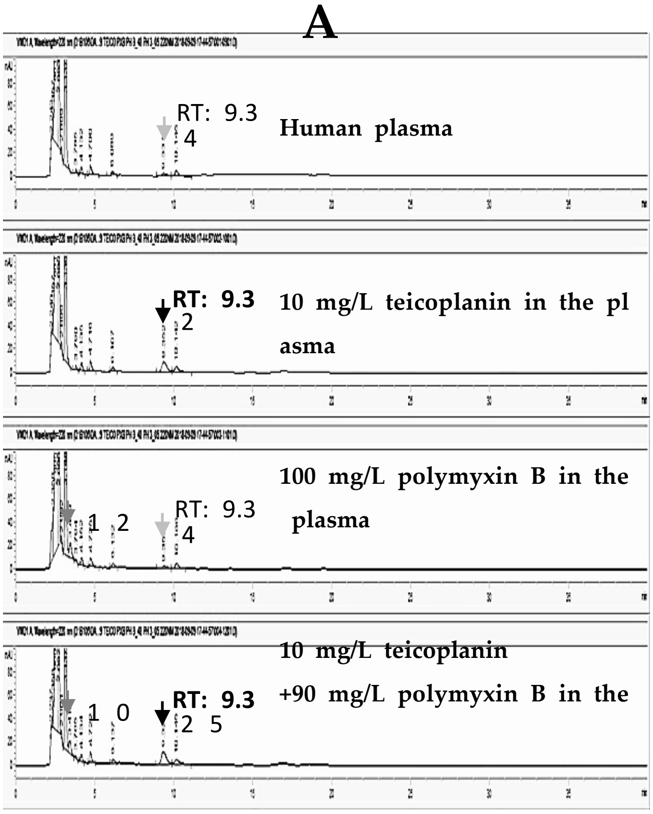

B

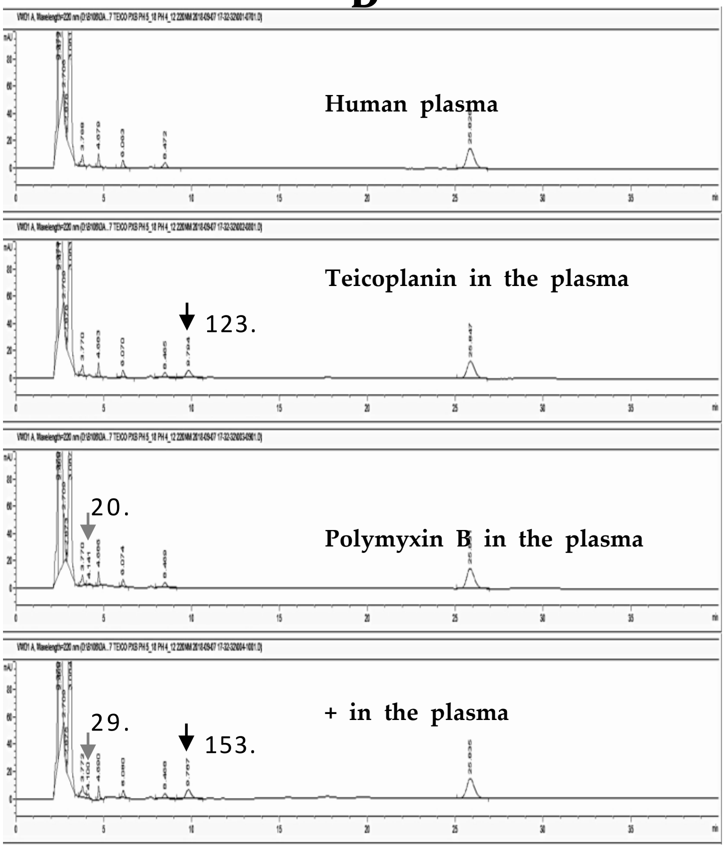
Human plasma
Teicoplanin in the plasma
123.
20.
Polymyxin B in the plasma
29.
153.
+ in the plasma

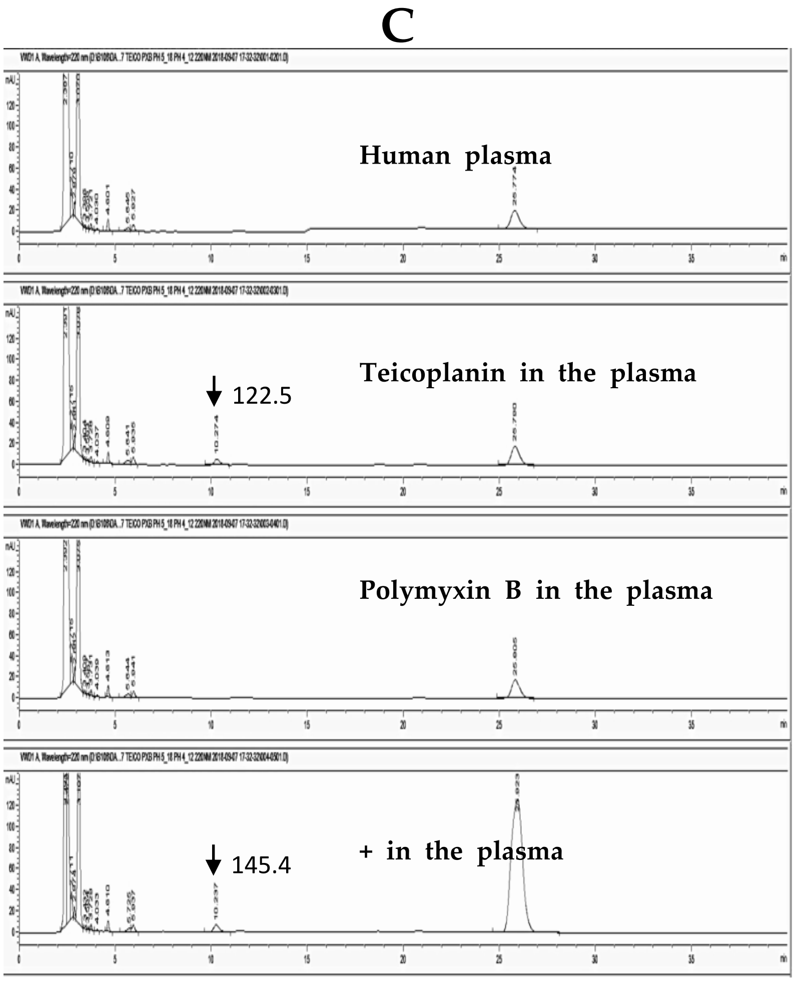

Figure S2. The chromatogram of the elution pattern by different pH conditions at 220 nm detection. (A) pH 3.0, (B) pH 4.12 and (C) pH 5.17 of the mobile phase (10 mM NaH_2PO_4:ACN:MeOH=70:25:5 (*v*/*v*/*v*)). (A) A2-2 was overlapped with plasma peak. (B) polymyxin B1 peak area was decreased. (C) polymyxin B1 peak was disappeared. Black arrows, grey arrows and light grey arrows indicated A2-2, B1 and the plasma endogenous peak, respectively. Numerical values represented the indicated peak area.

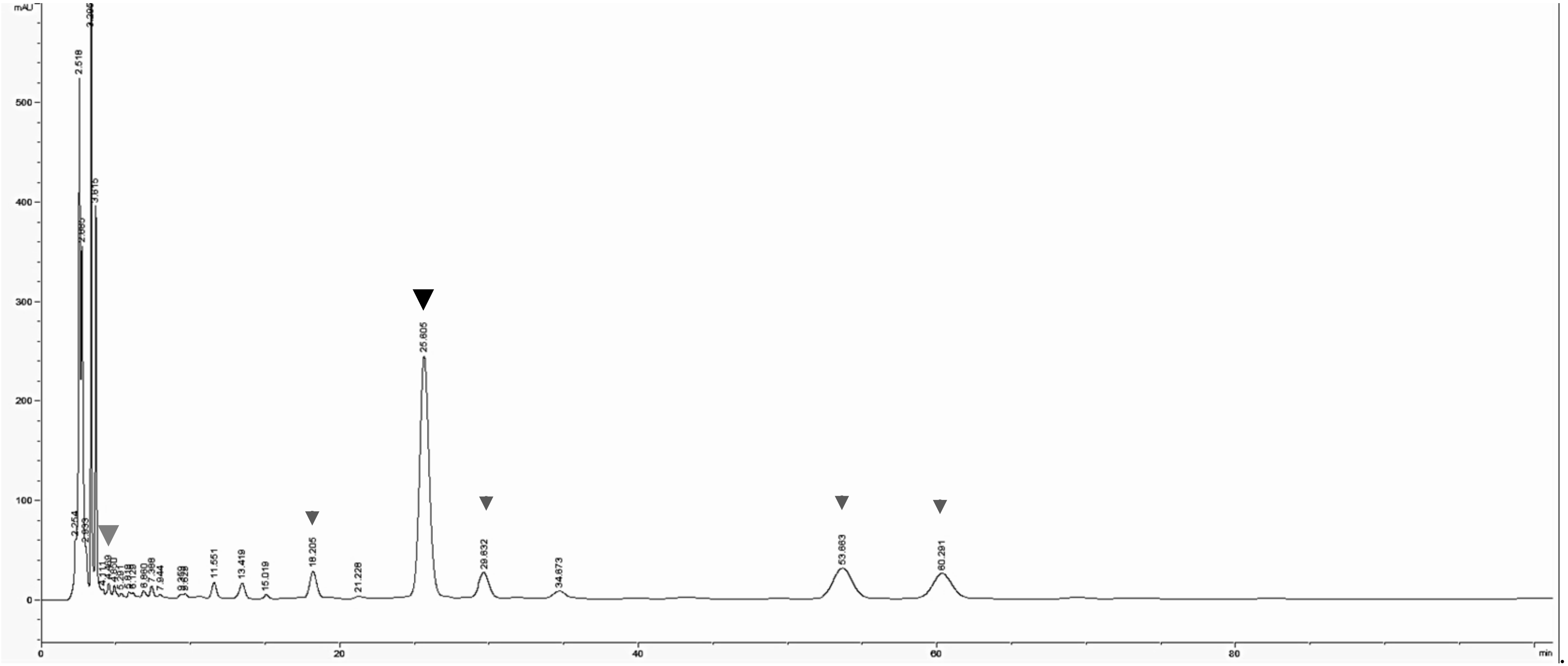

Figure S3. The chromatogram of the elution of teicoplanin with IS with 10 mM NaH_2PO_4:ACN=78:22 (*v*/*v*), pH 2.0, at 220 nm detection. Light grey arrow indicated IS (polymyxin B2) peak. Black arrow and 4 dark grey arrows indicated the peaks teicoplanin A2-2 and other isoforms, respectively.

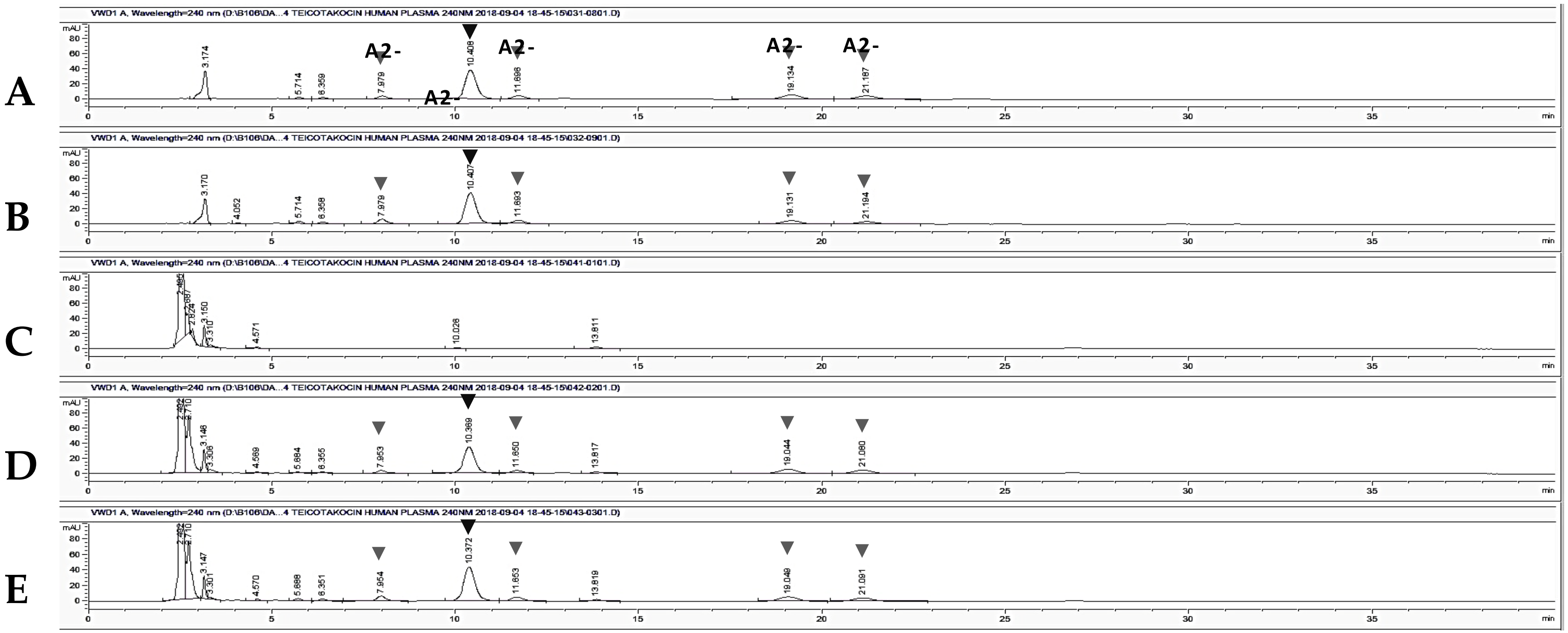

Figure S4. The chromatogram of the elution of teicoplanin (Sigma) and Tapocin® injection (HK innoN) (10 mM NaH_2PO_4:CAN:methanol=70:25:5 (*v*/*v*/*v*), pH 2.0, 240 nm detection). (A) 100 µg/mL of teicoplanin. (B) 100 µg/mL of Tapocin® injection. (C) Human plasma blank (Sigma). (D) 100 µg/mL of teicoplanin in plasma. (E) 100 µg/mL of Tapocin® injection in plasma. Light grey arrow indicated IS (B2) peak. Black arrow, and 4 dark grey arrows indicated the peaks teicoplanin A2-2 and other isoforms, respectively.

pharmaceutics

MDPI

Table S1. Stability of teicoplanin (A2-2) and IS (polymyxin B2) (n = 3).

**Compound (Concentration)**	**Recovery (%)**	**4℃, 5-D (%)**	**Post-preparation, 7-D (%)**
Teicoplanin A2-2 (L)		100.96 ± 11.32	108.56 ± 24.10
Teicoplanin A2-2 (M)	104.67 ± 14.75		105.94 ± 2.00
Teicoplanin A2-2 (H)		101.84 ± 1.59	102.58 ± 7.60
IS B2 (100 mg/L)	98.47 ± 6.09	99.34 ± 3.82	

D, day; L, 7.81 mg/L; M, 62.5 mg/L; H, 500.0 mg/L; the values of accuracy, mean ± S.D. a, post-preparation of teicoplanin and IS mixture.
